# Improving the reporting of intervention studies underpinned by a systems approach to address obesity or other public health challenges

**DOI:** 10.3389/fpubh.2022.892931

**Published:** 2022-07-26

**Authors:** Bai Li, Steven Allender, Boyd Swinburn, Mohammed Alharbi, Charlie Foster

**Affiliations:** ^1^School for Policy Studies, Centre for Exercise, Nutrition and Health Sciences, University of Bristol, Bristol, United Kingdom; ^2^Faculty of Health, Global Obesity Centre (GLOBE), Institute for Health Transformation, Deakin University, Geelong, VIC, Australia; ^3^School of Population Health, University of Auckland, Auckland, New Zealand

**Keywords:** reporting guidance, systems approach, obesity, public health intervention, system thinking, intervention development, intervention evaluation, intervention implementation

## Introduction

A systems approach to obesity prevention is increasingly urged (1, 2). However, confusion exists on what a systems approach entails in practice, and the empirical evidence on this new approach is unclear. Several reviews (3–6) have tried to synthesize available evidence on a systems approach targeting obesity and other public health areas, but found that authentic, comprehensive application of this approach is scarce. We believe this is largely due to the uncertainty around the exact meaning of “a systems approach,” and sub-optimal reporting.

Fully and transparently reported evidence can improve our understanding of how a systems approach is applied practically in different cultures and settings, support methodological development, and improve synthesis of emerging evidence on the effectiveness of this new approach.

## Recommended questions to guide the reporting and review of future work

As a team of experts who have advocated and applied systems science to address obesity and other public health challenges with ongoing empirical studies across 16 countries, we developed a list of practical questions to assist academic authors, journal editors and other interested stakeholders to design, report or review future interventions that apply an authentic systems approach to tackle obesity or other public health challenges. These questions were developed based on the latest academic knowledge, and comparative reflection on what were (or were not but self identified as) an authentic application of a systems approach published in public health journals (submitted to the collection of this journal).

Questions are organized by the three broadly defined and interrelated stages of an intervention's life cycle: “development,” “implementation/delivery” and “evaluation.” It is important to note that by “intervention(s),” we refer to interdepended programme of work containing multiple, coordinated actions aimed to stimulate, sustain, or re-orientate systemic changes. Moreover, in practice, the process of developing, implementing/delivering, and evaluating any complex intervention should be continuous, iterative and reflective (7). In a systems approach, the main stages of the intervention's life cycle may occur simultaneously (e.g., continuous monitoring and responding to changes while implementing agreed systemic actions).

### Intervention development

1. Have the authors clearly defined the public health problem being addressed?
2. Have the authors specified the theoretical underpinning of the systems approach (e.g., System Dynamics) applied to develop the intervention and justified their choice? *Simply saying the intervention was developed using a systems approach is not sufficient*.
3. Have the authors specified the methods (e.g., Group Model Building) applied to develop the intervention and justified their choice? *Simply saying the intervention was developed using a systems approach is not sufficient*.
4. Have the authors made any adaptations or methodological innovations to the referred development process to suit local settings or cultures?
5. If the answer to the 4^th^ question is YES, have the authors described such changes in sufficient detail to support methodological learning and advancement?
6. Have the authors clearly defined the targeted intervention community for each intervention in terms of its geographic/authoritative boundaries as well as the size and characteristics of the targeted population?
7. Have the authors described the environment (physical, cultural, socio-economic, and policy environments) within which the intervention was developed with sufficient detail to allow the readers to understand the development context? *Among others, this should include existing interventions/policies and how the local government and key stakeholders viewed the public health problem being addressed*.
8. Have the authors described in sufficient detail the process of identifying and choosing key subsystems/organizations/partners/decision-makers within the system prior to approaching them to develop a collective understanding of the system?
9. Have the authors described in sufficient detail the process of gaining support from senior leaders of those subsystems/organizations prior to developing a collective understanding of the system?
10. Have the authors described the subsequent steps involved in the intervention development process in sufficient detail? *To answer this question, consider whether the authors provided methodological information related to participants (and other individuals), activities/process, locations, duration, outputs, instruments, and materials? A flowchart is recommended in addition to the description in the text*.

### Intervention delivery/implementation

1. Have the authors clearly defined each intervention community (if multiple communities/cities/regions were included in the study/project) in terms of the geographic/authoritative boundaries as well as the size and characteristics of each beneficiary population?
2. Have the authors described the intervention environment (physical, cultural, socio-economic, and political environments) of each intervention community/city/region with sufficient detail to allow the readers to understand the intervention context? *Among others, this should include existing interventions/policies and how the local government and key stakeholders viewed the public health problem being addressed*.
3. Have the authors specified who were involved in the delivery of jointly identified and prioritized intervention actions and their responsibilities?
4. Have the authors specified the responsibilities of all individuals and organizations involved in the delivery of jointly identified and prioritized intervention actions?
5. Have the authors described with sufficient detail how communication and aligned collective actions across diverse action groups/stakeholders were maintained and monitored?
6. Have the authors described how to ensure a shared feeling of joint ownership (of the intervention) across diverse stakeholders or action groups?
7. Have the authors described in sufficient detail what were delivered/implemented, including the initial plan and subsequent changes to the initial plan?
8. If any intervention actions were adjusted, re-designed or terminated in response to results of ongoing intervention monitoring or other causes (e.g., lack of funds or change of leadership), have the authors explained the reasons for such changes?
9. Have the authors reported the challenges/barriers and facilitators to deliver the intervention?
10. Have the authors described the nature/sources of funding allocated to support the interventions?

### Intervention monitoring and evaluation

1. Have the authors defined the overall evaluation approach (e.g., stepped wedge design, natural experiment or routine data collection)?
2. Have the authors discussed how the chosen evaluation approach reflects features of systems thinking (e.g., complexities and dynamics)? *Following considerations may help to answer this question:*
  - ∙ *Have the authors described and justified methods used to assess how individual intervention actions worked together, interacted with each other and generated changes to the whole system?*
  - ∙ *Was ongoing monitoring of intervention impact included as part of the overall evaluation work (in addition to endpoint outcome measures)?*
  - ∙ *Have the authors measured and reported on unintended consequences? If yes, have they reported the methods and results with sufficient detail?*
  - ∙ *Have the authors described any attempt to understand how the system evolves over time?*
3. Have any of the evaluation outcomes been used to review and update stakeholders' understanding of the system gained prior to intervention delivery?
4. If the answer to the above question is YES, have the authors described what, when and how ongoing evaluation outcomes were used to support intervention delivery/implementation?
5. Have the authors reported on the challenges/barriers and facilitators to evaluation of intervention impact?
6. If the authors adapted/amended an existing evaluation approach/method or invented new methods, have these adaptations/innovations been described with sufficient detail to support methodological learning and advancement?
7. Have the authors described in sufficient detail what and when impact indicators/outcomes were measured and how?
8. If process and economic evaluations were included in a study/project, have the authors described the evaluation approach and methods in sufficient detail (within the same publication or elsewhere)?
9. If methodological adaptations or innovations were made to traditional process/economic evaluation approaches, have the authors described their approaches and methods in sufficient detail to support methodological learning and advancement?
10. Have the authors provided other information on study/project results (with reference to established reporting guidance if available) to allow readers to understand and assess results?
11. If any, have the authors identified, recorded, and reported major changes in the intervention environment (e.g., natural disasters, new public health crises and changes of national policies relevant to the public health problem being addressed) during the intervention delivery/implementation period that might influence accurate evaluation of the intervention outcomes?
12. If the answer to the 11^th^ question is YES, have the authors discussed the potential impacts of those major changes in the intervention environment to help readers interpret the reported intervention results?

## Discussion

This Opinion paper presents the first guidance for reporting public health interventions underpinned by a systems approach in the format of practical questions essential to intervention development, delivery/implementation and evaluation.

These questions will help researchers, editors, reviewers and policy makers to pay attention to, record and report information that have often been ignored in current practice but are valuable for the methodological advancement in this field. For example, we encourage authors to fully report contextual/cultural adaptations in applying a systems approach. We also ask authors to report any methodological adaptations or innovations made to traditional process/economic evaluation approaches. Moreover, unlike many interventions delivered in a controlled setting or design, the intervention context and setting in any system-level interventions is dynamic and constantly changing. Therefore, we encourage authors to identify, record and report major changes in the intervention environment (e.g., natural disasters, new public health crises and changes of national policies relevant to the public health problem being addressed) during the period of intervention delivery/implementation.

Some of the reporting suggestions are unique to complex, systemic public health interventions, and so have not been included in existing reporting guidance developed for general trial studies. For instance, we ask authors to report whether any evaluation outcomes have been used to review and update the system map drawn previously? We encourage authors to discuss how their chosen intervention evaluation approach reflects features of systems thinking (e.g., complexities and dynamics). Several hint questions are also offered to assist this (e.g., have the authors described any attempt to understand how the system evolves over time?).

We hope these questions can assist the design, reporting and reviews of future public health interventions applying an authentic systems approach, and provide the first step toward developing a comprehensive reporting guidance for systemic interventions in public health. We welcome academic peers, journal editors and policy makers to share their thoughts about these questions, collectively making the first step toward developing a comprehensive guidance for reporting public health interventions underpinned by a systems approach.

## Author contributions

BL conceived the idea, provided the first draft of the manuscript, and wrote the guidance. SA, BS, and CF edited the manuscript. All authors read and approved the final version of the manuscript.

## Funding

This paper was an output from the SYSTAM CHINA SEACS project funded by the UK Medical Research Council (grant number: MR/V004174/1).

## Conflict of interest

The authors declare that the research was conducted in the absence of any commercial or financial relationships that could be construed as a potential conflict of interest.

## Publisher's note

All claims expressed in this article are solely those of the authors and do not necessarily represent those of their affiliated organizations, or those of the publisher, the editors and the reviewers. Any product that may be evaluated in this article, or claim that may be made by its manufacturer, is not guaranteed or endorsed by the publisher.
